# The perceived impact of the COVID-19 pandemic on child protective services in Saudi Arabia

**DOI:** 10.3389/fpubh.2026.1749813

**Published:** 2026-03-06

**Authors:** Majid Aleissa, Norah Alhowaish, Wseemah Almutairi, Abdulaziz S. Alangari, Naila A. Shaheen, Rania Abdelhamid, Anna Rita Ronzoni, Dalia Gaber Sos Boles, Hala Sakr

**Affiliations:** 1 King Abdullah International Medical Research Center, Riyadh, Saudi Arabia; 2 King Saud bin Abdulaziz University for Health Sciences, Riyadh, Saudi Arabia; 3 King Abdullah International Medical Research Center, Ministry of National Guard - Health Affairs, King Saud bin Abdulaziz University for Health Sciences, Riyadh, Saudi Arabia; 4 World Health Organization Regional Office for the Eastern Mediterranean, Cairo, Egypt; 5 Department of Community Medicine, Faculty of Medicine, Ain Shams University, Cairo, Egypt

**Keywords:** child protective services, COVID-19, emergency preparedness, Saudi Arabia, violence against children

## Abstract

**Background:**

The COVID-19 pandemic has greatly impacted the child protective services (CPS) globally, resulting in increased risks of violence against children (VAC). This study aims to assess the perceived impact of COVID-19 on CPS operations by CPS professionals from health, social, law enforcement, and educational sectors in Saudi Arabia.

**Methods:**

A cross-sectional survey was conducted between September 2021 and June 2022 among 257 CPS professionals from health, social, law enforcement, and educational sectors in Saudi Arabia. The questionnaire, developed with oversight by WHO, covered nine domains: violence trends, reporting and documentation, response and follow-up, legislation, resources, staff preparedness, prevention programs, knowledge, and challenges. Quantitative data were analyzed using descriptive statistics and Cramér’s V tests, while qualitative data were examined thematically.

**Results:**

Health sector professionals more frequently reported marked increases in the number of reported VAC cases and their severity compared to non-health participants. The results have shown limited change in reporting systems, with the exception of a few newly introduced mechanisms. Staff training, crisis preparedness, and digital infrastructure were insufficient, with limited remote follow-up. Qualitative findings highlighted family stress, financial hardship, and social isolation as risk factors. Major challenges included reduced case accessibility, weak inter-agency coordination, and inadequate protective measures.

**Conclusion:**

The study, conducted in the context of the COVID-19 pandemic in Saudi Arabia, reveals the need to further develop some aspects of CPS emergency preparedness. Strengthening intersectoral collaboration, investing in digital tools, enhancing staff training, and promoting community awareness are essential to ensure the resilience of CPS during similar future situations.

## 1 Introduction

The COVID-19 pandemic has exacerbated global disruptions and caused far-reaching consequences across multiple sectors, including the well-being and safety of children (1).

Even before the pandemic, violence against children (VAC) was highly prevalent; a pre-pandemic meta-regression study reported prevalence rates of physical and psychological violence and witnessing interparental violence for both boys and girls from the age of 2 to 14 years to be greater than 50% (2). According to a review conducted in the context of the pandemic between the years 2020 and 2022, there was an obvious decline in the reporting of VAC, yet an increase in severe cases of child maltreatment during the pandemic compared to the pre-pandemic era (3).

There are several factors related to quarantine measures that increase the risk of VAC, including heightened household stress, economic insecurity, job loss, social isolation, and prolonged confinement. At the same time, the capacity to identify children at risk was substantially weakened. Professionals who typically play a frontline role in recognizing abuse, such as teachers, childcare workers, healthcare providers, and social workers, had limited contact with children due to lockdowns and the widespread shift to virtual schooling (4). These disruptions affected not only families but also the intersectoral systems designed to protect children.

Evidence from the Eastern Mediterranean Region further highlights these challenges. A comparative review of media reports showed a marked increase in physical maltreatment among adolescents aged 11–17 years during the pandemic, rising from 14% in 2019 to 32% in 2020. In contrast, reported incidents among children aged 5–10 years declined from 28% in 2019 to 23% in 2020 as the lockdown prevented frontline providers in the community (including teachers, social workers, nurses, and physicians, who would be able to detect abuse in normal circumstances) from reporting suspected abuse. Overall, 72% of physical maltreatment occurred among male children, and there was a significant increase in incidents among female children, from 9% in 2019 to 33% in 2020 (5).

The response systems for violence against children in the Gulf Cooperation Council (GCC) countries (Bahrain, Kuwait, Oman, Qatar, Saudi Arabia, and the United Arab Emirates) were suboptimal during COVID-19 (6–12). The sudden onset of the crisis led to rapid changes in service delivery models, uneven preparedness, and variability in response capacity both between and within countries (13–16). Child Protective Services (CPS), which plays a central role in safeguarding children exposed to violence, was among the services most affected (17).

In Saudi Arabia, the CPS aims to protect children from all forms of abuse, neglect, and violence in accordance with the Child Protection Law and international conventions. Their responsibilities include receiving and investigating reports, assessing risks, coordinating with health, education, and judicial sectors, ensuring immediate child safety, and providing family support or alternative care through the kafala system when necessary (18, 19). The effectiveness of these functions depends heavily on intersectoral coordination and continuous access to children, both of which were challenged during the pandemic.

Effective prevention of and response to VAC requires strong integration between social and healthcare systems. Saudi Arabia has emphasized the value of integrating mental health and psychosocial support services into primary healthcare through strengthened collaboration between health, social, and community-based services. These integrated approaches are essential to ensure timely identification, referral, and follow-up of cases, and to support the effective functioning of the system through coordinated, multisectoral responses (20). Evidence from other settings, such as the Italian experience, demonstrates how structured coordination between social and health services, supported by multidisciplinary collaboration, can enhance continuity of care for vulnerable populations and strengthen system resilience during public health emergencies (21, 22). This highlights the importance of integrated care models and continuity of services in enhancing child protection systems.

Despite the importance of these systems, limited empirical research has examined how the COVID-19 pandemic affected CPS operations in GCC countries, particularly from the perspective of professionals directly involved in child protection. The present study aims to examine how the COVID-19 pandemic affected Child Protective Services in Saudi Arabia, focusing on perceived changes in service delivery, intersectoral coordination, and professional practice across health and non-health sectors. Specifically, the study explores how remote work arrangements, service disruptions, and coordination challenges influenced CPS functions during the pandemic. This study is part of a broader collaboration between the National Family Safety Programme (NFSP), the WHO Collaborating Centre for Child Maltreatment Research and Planning, and the WHO Eastern Mediterranean Regional Office.

## 2 Methods

### 2.1 Study design

A cross-sectional study design was conducted during the period from September 1st, 2021 to June 22nd, 2022, through an online survey, which was distributed to the participants/respondents. The extended survey period was designed to capture data across the three waves of the COVID-19 pandemic in Saudi Arabia and to accommodate varying institutional approval timelines across different governmental organizations. All responses received from targeted participants meeting the inclusion criteria were included in the analysis.

### 2.2 Participants

Professionals working at CPS in KSA were invited to participate in the study through an invitation email explaining the nature and aim of the study, in which participants were also assured that their participation was entirely voluntary. Upon receiving their agreement, participants were asked to sign an informed consent form electronically. Afterward, the link for the web-based survey was sent to them, along with the investigators’ contact information for any questions that may arise.

#### 2.2.1 Sampling frame and recruitment

A purposive sampling approach was used to identify professionals working in child protection services (CPS) across five key sectors in the Kingdom of Saudi Arabia. The National Family Safety Program (NFSP), officially contacted designated organizations in each sector and formally requested the email addresses and contact information of employees whose roles involve working with children, either directly or indirectly.

#### 2.2.2 Inclusion criteria

Professionals of any age or sex working at health facilities, educational institutions, law enforcement and judicial sectors, as well as non-profit organizations that deal with violence against children (VAC) in Saudi Arabia, were eligible to participate.

#### 2.2.3 Exclusion criteria

Professionals practicing outside the Kingdom of Saudi Arabia at the time of data collection. Retired or unemployed individuals during the study period were excluded. Other from that All professionals working in child protection-related roles across the targeted sectors who received the survey invitation and provided informed consent were eligible to participate, regardless of their years of experience, educational background, or specific job title.

#### 2.2.4 Survey distribution

A total of 721 professionals across all sectors received an invitation email explaining the nature and aim of the study. Upon receiving their agreement, participants were asked to electronically sign an informed consent, after which the link for the web-based survey was sent to them, including the investigators’ contact information for any questions that may arise.

#### 2.2.5 Response rate and sample composition

By June 2nd, 2022, a total of 257 responses were received, yielding an overall response rate of 35.6%. The distribution varied by sector: the health sector received the highest number of invitations (*n* = 187) with 113 responses (60.4% response rate), followed by the social sector with 315 invitations and 73 responses (23.2% response rate). The educational sector received 76 invitations with 16 responses (21.1% response rate), while the law enforcement/judicial sector received 93 invitations, yielding 30 responses (32.3% response rate). Other sectors received 50 invitations with 25 responses (50.0% response rate).

### 2.3 Study tools

A self-administerative questionnaire was developed and reviewed by the WHO Collaborating Center (The National Family Safety Program), with oversight by the WHO Eastern Mediterranean Regional Office. The WHO contributed to the conceptualization and design of the study and provided technical input throughout the research process. WHO co-authors participated in the revision of the study tool, interpretation of findings, and critical revision of the manuscript.

The Survey converted into an electronic format by using LimeSurvey (Version 4.1.6 + 200,220). The survey’s skeleton is based on 9-dimensions to assess the perceived impact of COVID-19 pandemic on CPS: (1) Trends of violence (4 items); (2) Reporting and documentation (8 items); (3) Response and follow up (4 items); (4) Procedures and legislations (3 items); (5) Human and material resource (5 items); (6) Staff preparedness and work environment (6 items); (7) Prevention programs implementation (7 items); (8) Professionals’ scientific knowledge (4 items); and (9) Challenges and recommendations (2 items). The questionnaire included closed-ended questions as well as open-ended questions to enable the participants to clarify their answers, especially in the challenges and recommendations section. (see the attached questionnaire). Cronbach’s alpha was used to test the reliability of the different sections of the questionnaire. All scales have alpha coefficients between 0.67 and 0.85.

### 2.4 Ethical consideration

Ethical approval was obtained from the Institutional Review Board at King Abdullah International Medical Research Center (KAIMRC), NRC21R.039.02. Riyadh, Saudi Arabia.

### 2.5 Data analysis

#### 2.5.1 Quantitative analysis

Descriptive statistics were calculated using mean ±SD for continuous variables, and frequencies and percentages for categorical variables. Cramér’s V was used to estimate the effect size computed by SPSS v. 8.0 (23) to determine the strengths of the associations between the two groups of participants and their responses to the eight dimensions of the survey. The interpretation of Cramér’s V was ≤ 0.2 = “weak” whilst 0.21 to 0.6 = “moderate” and > 0.6 = “strong” [(23), p.1]. *p*-values of <0.05 were considered significant.

#### 2.5.2 Qualitative analysis

For the coding and analysis of the qualitative data, an inductive thematic analysis approach was applied, whereby codes were developed directly from the data based on participants’ responses (24). Two researchers independently coded the transcripts, after which a third researcher reviewed the coding to ensure consistency and identify any discrepancies. The final codes were then organized into four overarching themes, corresponding to the four open-ended questions (24).

## 3 Results

### 3.1 Quantitative findings

Out of 257 participants, 56.4% were females, and 43.6% were male The mean age of the participants was 39.9 ± 8.0 years, whereas 40.5% of participants were aged 40–49 years, and less than 2% were aged ≥60 years (Table 1).

**Table 1:** Demographic profiles of participants (*N* = 257).

Variable	Number (%)
Age (mean±SD)	39.9 ± 8.0
Age categories	
<30 years	22 (8.6)
30–39 years	102 (39.7)
40–49 years	104 (40.5)
50–59 years	24 (9.3)
≥ 60 years	5 (1.9)
Sex	
Male	112 (43.6)
Female	145 (56.4)
Employee’s organization	
Government	231 (89.9)
Non-Government	26 (10.1)
Employee’s area of operation	
Local (city, province or emirate level)	172 (66.9)
National	85 (33.1)
Employee’s sector	
Health	113 (44.0)
Social	74 (28.8)
Law Enforcement/Judicial	45 (17.5)
Educational	18 (7.0)
Others (administrative, human resources)	7 (2.7)

Regarding participants’ opinion in violence trends during pandemic, respondents from the health sector reported a marked increase in both the number (24.8%) and severity (22.1%) of cases, while in non-health report being unsure about trends, especially in the number of cases (24.3%). The mode responses differed between sectors with no significant (Table 2).

**Table 2:** Comparison of trends of violence against children described by the health sector (*n* = 113) vs. non-health sector (*n* = 144) during the COVID-19 pandemic in Saudi Arabia.

Item	Sector	Marked increase *n* (%)	Slight increase *n* (%)	No change *n* (%)	Not sure *n* (%)	Slight decrease *n* (%)	Marked decrease n (%)	Mode	Cramer’s V	*p*-value
Number of cases of violence against children registered during the pandemic.	Health	28 (24.8)	18 (15.9)	18 (15.9)	18 (15.9)	14 (12.4)	17 (15.1)	Marked increase	0.18	0.16
	Non-Health	31 (21.6)	35 (24.3)	15 (10.4)	35 (24.3)	13 (9.0)	15 (10.4)	Not Sure, Slight increase		
Severity of cases of violence against children recorded during the pandemic.	Health	25 (22.1)	22 (19.5)	22 (19.5)	19 (16.8)	12 (10.6)	13 (11.5)	Marked increase	0.20	0.08

Participants across both health and non-health sectors reported minimal positive impact on reporting mechanisms. The majority noted ‘no impact’ with no significant. For documentation, both sectors reported no impact, with significant difference between the two groups with small effect size. Most participants reported that no new reporting mechanisms or new documentation systems were developed (*p* < 0.01) (Table 3).

**Table 3:** Comparison of reporting and documentation on violence against children described by the health sector (*n* = 113) vs. non-health sector (*n* = 144) during the COVID-19 pandemic in Saudi Arabia.

Item	Sector	Positive impact *n* (%)	Negative impact *n* (%)	Both *n* (%)	No impact *n* (%)	Not sure *n* (%)	Mode	Cramer’s V	*p*-value
Impact of Covid-19 pandemic on mechanisms and means of reporting violence	Health	19 (16.8)	14 (12.4)	7 (6.2)	50 (44.3)	23 (20.3)	No impact	0.12	0.57
	Non-Health	24 (16.7)	16 (11.1)	9 (6.3)	53 (36.8)	42 (29.1)	No impact		
Impact of Covid-19 pandemic on mechanisms/records for documenting violence	Health	9 (8.0)	11 (9.7)	3 (2.7)	74 (65.5)	16 (14.1)	No impact	0.24	***<0.01***
	Non-Health	12 (8.3)	12 (8.3)	3 (2.1)	67 (46.6)	50 (34.7)	No impact		

Item	Sector	Yes *n* (%)	No *n* (%)	Not applicable *n* (%)	Mode	Cramer’s V	*p*-value
New reporting mechanisms were put in place	Health	10 (8.9)	89 (78.7)	14 (12.4)	No	0.24	***<0.01***
	Non-Health	26 (18.0)	80 (55.6)	38 (26.4)	No		
Mechanisms/documentation of records were created for violence against children	Health	10 (8.9)	84 (74.3)	19 (16.8)	No	0.22	***<0.01***
	Non-Health	16 (11.1)	77 (53.5)	51 (35.4)	No		

*Bold and italic values indicate statistically significant results at *p* < 0.05.

Table 4, both health and and non health sectors reflected that responses and follow-up mechanisms were largely uninterrupted during the pandemic. Within health sector, mechanisms ‘never stopped’, compared to non-health sector. Most participants indicated no new follow-up systems were developed with *p* value <0.01. Few legislative changes were reported, and most confirmed no new procedures with *p* = 0.04.

**Table 4:** Comparison of response mechanisms, follow-up mechanisms, procedural protocols, and legislative measures addressing violence against children described by the health sector (*n* = 113) vs. non-health sector (*n* = 144) during the COVID-19 pandemic in Saudi Arabia.

Item	Sector	Completely *n* (%)	Temporary *n* (%)	Never stopped *n* (%)	Not applicable *n* (%)	Mode	Cramer’s V	*p*-value
Response and follow-up mechanisms for cases of violence against children stopped	Health	3 (2.7)	3 (2.7)	98 (86.7)	9 (8.0)	Never stopped	^a^	
	Non-Health	2 (1.4)	10 (6.9)	109 (75.7)	23 (16.0)	Never stopped		

Item	Sector	Yes *n* (%)	No *n* (%)	Not applicable *n* (%)	Mode	Cramer’s V	*p*-value
Developed new response and follow-up mechanisms for cases of violence	Health	12 (10.6)	78 (69.0)	23 (20.4)	No	0.20	***<0.01***
	Non-Health	25 (17.4)	70 (48.6)	49 (34.0)	No		

Item	Sector	Yes *n* (%)	No *n* (%)	Not sure *n* (%)	Mode	Cramer’s V	*p*-value
Procedures or legislation dealing with violence against children were introduced	Health	4 (3.5)	83 (73.5)	26 (23.0)	No	0.16	***0.04***
	Non-Health	8 (5.6)	84 (58.3)	52 (36.1)	No		
Procedures or legislation dealing with violence against children were withheld	Health	0 (0.0)	92 (81.4)	21 (18.6)	No	^a^	
	Non-Health	4 (2.8)	105 (72.9)	35 (24.3)	No		
Procedures or legislation contributed toward an increase in violence against children	Health	3 (2.7)	81 (71.7)	29 (25.7)	No	0.06	0.66
	Non-Health	7 (4.9)	100 (69.4)	37 (25.7)	No		

a Cannot determine *p*-value because of small cell size. *Bold and italic values indicate statistically significant results at *p* < 0.05.

In Table 5, higher proportion of workers in the health sector reported their organizations “always” provided preventive precautions compared to those in the non-health sector. Only 10.6% of health workers reported complete conversion to remote work. In contrast, 30.6% of non-health workers reported full remote conversion with significant difference *p* < 0.001.

**Table 5:** Comparison of staff preparation and work environments of child protection workers by the health sector (*n* = 113) vs. non-health sector (*n* = 144) during the COVID-19 pandemic in Saudi Arabia.

Item	Sector	Never *n* (%)	Rarely *n* (%)	Sometimes *n* (%)	Often *n* (%)	Always *n* (%)	Not applicable *n* (%)	Mode	Cramer’s V	*p*-value
Your organization provided workers with preventive precautions to protect against infection during the pandemic.	Health	2 (1.8)	3 (2.7)	9 (8.0)	15 (13.3)	74 (65.5)	10 (8.8)	Always	^a^	
	Non-Health	8 (5.6)	5 (3.5)	13 (9.0)	32 (22.2)	69 (47.9)	17 (11.8)	Always		
Your organization converted the work environment to virtual (remote working).	Health	12 (10.6)	72 (63.7)	29 (25.7)				Partially	0.25	***<0.001***
	Non-Health	44 (30.6)	78 (54.2)	22 (15.3)				Partially		

a Cannot determine *p*-value because of small cell size.

Regarding the material and human resources allocated to child protection, It was reported that the pandemic had limited the impact on CPS resources. For material resources, 54.0% of health and 52.8% of non-health respondents reported ‘no impact’. For human resources, 65.5% (health) and 56.9% (non-health) also reported no change. Governmental sources were the primary funders (Health: 77.0%, Non-Health: 83.3%).

According to respondents’ perceptions of the effectiveness of international and local efforts in raising knowledge about VAC, Most participants believed that efforts had enhanced the knowledge about VAC. Specifically, 36.58% of respondents agreed that international initiatives contributed to raising awareness, while 52.14% believed it helped to some extent. Similarly, 37.74% of respondents felt that local efforts had a clear impact, and 50.19% reported these efforts were helpful to some extent. Notably, a slightly higher percentage of respondents viewed local efforts positively compared to international ones. The findings suggest a slightly stronger endorsement of local efforts.

Participants’ opinion regarding the contribution of local and international efforts in raising the knowledge on VAC, the majority in both sectors agreed, to some extent, that such international and local efforts enhanced knowledge. 47.8% of health sector respondents and 55.6% from the non-health sector believed international organizations contributed “to some extent,” while 49.6 and 50.7%, respectively, gave the same response regarding local institutions. The mode response in all groups was “Yes, to some extent” with no significant difference between sectors.

### 3.2 Qualitative findings

Qualitative data were analyzed under four themes to compile the participants’ responses.

#### 3.2.1 Theme 1: risk factors contributing to violence against children during the pandemic

Participants identified several key risk factors that intensified violence against children during the COVID-19 pandemic. A common factor was disrupted family communication, described as parents’ limited interpersonal skills, neglect of children’s emotional needs, and increased isolation caused by excessive use of electronic devices.

Emotional distress within families emerged as another critical factor. Professionals cited addiction of one or both parents, heightened psychological pressures, and diminished religious or moral support as underlying issues. These stressors were further compounded by social disconnection, with some families experiencing isolation and a lack of awareness about child protection services or legal frameworks.

The role of financial hardship was also emphasized. Economic strain due to job loss or reduced income increased family tension and created circumstances where abuse was more likely to occur. Additionally, limited access to healthcare prevented families from seeking timely support, potentially delaying the identification and reporting of abuse.

#### 3.2.2 Theme 2: consequences of violence against children

Respondents reported a wide range of serious outcomes linked to child maltreatment. These included physical injuries and long-term disabilities, as well as mental health issues such as anxiety, depression, aggression, and social withdrawal.

Several participants described how children subjected to abuse developed low self-esteem and distorted self-image, which in severe cases led to suicidal ideation. Longitudinal concerns were also expressed: exposure to violence was seen as perpetuating a cycle of abuse, with abused children more likely to exhibit violent behaviors or become victims or perpetrators in adulthood.

#### 3.2.3 Theme 3: challenges in delivering child protection services during COVID-19

Respondents emphasized difficulty in conducting case assessments and follow-up due to lockdowns and restricted mobility, which limited their physical access to children and families.

In many organizations, the shift to remote work and limited staff availability has reduced operational efficiency. Participants also noted the absence of emergency protocols, which left staff uncertain about how to proceed in crisis conditions.

Communication breakdown, both within institutions and across agencies, was also identified as barriers to effective coordination. Professionals further expressed concern for their own personal safety, often working in high-risk environments without adequate protective measures or training in pandemic response.

#### 3.2.4 Theme 4: recommendations to improve child protection services during crises

A primary suggestion was to raise community awareness about child abuse, especially through targeted outreach during times of crisis.

Improving training for professionals on crisis response, including virtual assessment techniques and trauma-informed care, was seen as essential. Respondents also encourage increasing the investment in human and technological resources such as digital infrastructure.

Finally, stronger inter-agency collaboration and internal communication mechanisms were emphasized as critical to ensure continuity of care and reduce fragmentation across child protection networks.

**Table:** 

Themes	Codes
Theme 1: risk factors contributing to violence against children during the pandemic	Code 1 Communication within the family
	Code 2 Emotional stress during the pandemic
	Code 3 Limited interaction with the society
	Code 4 Financial stresses
	Code 5 Limited access to healthcare
Theme 2: consequences of violence against children	Code 1 Physical illnesses and disabilities
	Code 2 Psychological and behavioral disturbances
	Code 3 Low self-esteem and suicidal ideation
	Code 4 Death
	Code 5 Perpetuation of violence in future
Theme 3: challenges in delivering child protection services during COVID-19	Code 1 Reduce accessibility to cases (assessment and follow up) due to lock down/curfew
	Code 2 Reduce efficiency of human resources due to virtual/ limited working hours
	Code 3 Lack of guiding policies and procedures for the work during crises and pandemics
	Code 4 Insufficient intra and inter institutional communication
	Code 5 Provider’s concern about their personal safety
Theme 4: recommendations to improve child protection services during crises	Code 1 Promote CAN awareness particularly during crisis and pandemics
	Code 2 Enhance efficiency of human and technical resources
	Code 3 Train professionals on responding to CAN during crisis and pandemics
	Code 4 Improve collaboration between agencies and communication efforts within the organization

## 4 Discussion

This study provides critical insights into how CPS in Saudi Arabia were impacted by the COVID-19 pandemic as perceived by the professionals in the field. The findings reveal notable differences in how the health and non-health sectors’ professionals perceived and responded to VAC the COVID-19 pandemic in Saudi Arabia.

Our findings show that reporting and documentation mechanisms of VAC remained largely unchanged in Saudi Arabia during the pandemic. Most respondents indicated that no new systems were developed and that existing systems experienced little to no enhancement. These findings align with a retrospective observational study done in Birmingham, UK, which reported similar findings when assessing the effect of COVID-19 lockdown on child protection medical assessments, where a significant drop of 39% (95% CI 14 to 57%) in child protection and medical examination (CPME) referrals during 2020 compared with previous years with 78 referrals in 2018, 75 in 2019 and 47 in 2020 (25).

These findings are also in agreement with UNICEF’s Socio-economic Impact Survey of COVID-19 Response on CPS services, where out of 136 countries that responded, 104 countries reported a disruption in services related to violence against children. Around two-thirds of countries reported that at least one service had been severely affected, including South Africa, Malaysia, Nigeria, and Pakistan. South Asia, Eastern Europe, and Central Asia had the highest proportion of countries reporting disruptions in the availability of services (16).

Participants reported reduced access to cases, inefficient virtual follow-up, and a lack of crisis-specific training. This highlights a gap in emergency preparedness. As noted by Font et al. (26), the absence of a workforce continuity strategy within CPS systems globally left frontline workers often unsupported, despite being essential responders. Our findings underscore the need to embed staff wellbeing and resilience-building into future preparedness plans.

The current study pointed out the need for explicit policies to respond to the pandemic crisis, particularly in relation to remote assessments, interagency reporting, and legal obligations. Similarly, a study conducted in the U.S. on the effect of the pandemic on CPS found that many CPS workers operated in the absence of clear policy directives, relying instead on local leadership or professional discretion which lack of policy guidance resulted in operational inconsistencies and raised ethical concerns around child safety and staff accountability (27, 28). Kelly and Hansel (29) outlined the need for a plan to ensure that there are “eyes on” children. The lack of policy regarding what to do with parents who are experiencing a personal crisis that would not have occurred without the general crisis, and would not affect the context of care if it were not for the crisis, implies that the need for a collaborative multisectoral policy to ensure that the child’s right to protection is enforced.

The study results do not provide clear information on the existence of community-based child protection programming. Similarly, U.S. agencies experienced reduced visibility due to school closures and suspended in-person services, which act as a frontline for detecting abuse (27, 30). The qualitative open-ended questions in this study provided more in-depth elaboration on the factors that contribute to occurance of violence among children, the consequences of child maltreatment, and challenges facing child protection systems, and highlighted the recommendations to strengthen the child protection services.

Participants have identified disrupted family communication, parental emotional instability, and increased social isolation as key contributors to VAC during the pandemic. These findings mirror international evidence showing that caregiver stress, often exacerbated by remote schooling, confinement, and economic uncertainty, significantly increased children’s exposure to abuse and neglect during lockdowns (28, 31). Financial hardship, such as job loss or income reduction, further increases family tensions, consistent with global reports that link economic declines to elevated risks of domestic violence (32). Additionally, limited access to healthcare and psychosocial support services contributed to delayed detection and intervention (33).

Respondents, based on their perception, have highlighted a range of physical and psychological consequences linked to VAC, including anxiety, suicidal ideation, and behavioral issues. These are consistent with findings from both high-income and low-income contexts, which reported spikes in mental health issues among children during the pandemic (34).

Child protection professionals elaborated that they faced a number of operational challenges. Movement restrictions impaired face-to-face assessments and follow-ups, which is in agreement with many research studies that documented sharp declines in abuse reporting after school closures and social service limitations (35, 36). Institutional responses need to be supported by clear emergency protocols, adequate digital infrastructure, pandemic-specific training, and personal safety measures to enable appropriate remote interventions. These findings mirror those in global contexts, where CPS workers lacked PPE and mental health support despite working on the frontlines (37, 38).

Several limitations of this study should be acknowledged. First, the study relied on a questionnaire that was not based on a previously standardized tool; however, it was formalized by expert input, pilot-tested among a small sample of Arabic speakers, and subjected to reliability testing. This may limit comparability with findings from other contexts and warrants cautious interpretation of the results. Second, the use of self-reported surveys may introduce response and recall bias, particularly given the heightened stress experienced by professionals during the COVID-19 pandemic. Third, although the study included participants from both health and non-health sectors, the findings may not fully capture the diversity of experiences across all regions or service settings within Saudi Arabia, limiting generalizability.

Future research would benefit from the development and validation of standardized instruments tailored to assessing child protection system performance during emergencies, as well as from longitudinal designs that examine system adaptations and recovery beyond the acute phase of crises. Qualitative studies could further explore the mechanisms underlying intersectoral coordination challenges and the impact of remote working arrangements on professional practice. Additionally, comparative studies across countries or regions may provide valuable insights into best practices for strengthening integrated child protection responses during public health emergencies.

This study provides important evidence on how the COVID-19 pandemic affected CPS in Saudi Arabia, as perceived by professionals working across health and non-health sectors. In line with the study objectives, the findings demonstrate that while core protective measures and technical support mechanisms remained in place during the pandemic, significant challenges emerged in relation to training, service accessibility, intersectoral coordination, and crisis preparedness, particularly within the health sector. Differences in experiences between health and non-health professionals highlight variations in system readiness and adaptive capacity during emergency conditions.

The study underscores the critical importance of strengthening integrated child protection systems through targeted policy interventions, enhanced capacity building, and improved coordination between social and healthcare sectors. Expanding the accessibility of CPS professionals across multiple communication platforms and reinforcing referral pathways are essential to ensuring that all children in need of protection can be identified and supported during emergencies. These findings contribute to the growing evidence base on child protection system resilience and offer practical implications for improving preparedness and response to violence against children during public health emergencies and beyond.

## Data Availability

The original contributions presented in the study are included in the article/Supplementary material, further inquiries can be directed to the corresponding author.

## Author’s note

The opinions expressed in this article are those of the authors and do not reflect the official position of WHO. WHO takes no responsibility for the information provided or the views expressed in this article.
