# Supplementary material for: The perceived impact of the COVID-19 pandemic on child protective services in Saudi Arabia
Source: Front Public Health. 2026 Mar 6;14:1749813. doi: 10.3389/fpubh.2026.1749813 (PMC13003565; doi:10.3389/fpubh.2026.1749813)
Supplement: Supplementary file 1 [file Data_Sheet_1.pdf]

A Study on the Impact of COVID-19 on Violence Against Children Protection Services

Dear Participant:

The pandemic of COVID-19 had impacted many childhood services around the world including protection against violence. Therefore, the World Health Organization (WHO) East Mediterranean Regional Collaborative Center at the National Family Safety Program- Kingdom of Saudi Arabia is conducting a study in the Gulf Council Cooperation (GCC) countries to evaluate the impact of the pandemic on these services. The results of the study are expected to improve responses of organization in this or future crises.

As a professional working in an organization that delivers violence protection services to children in your country, you are invited to participate in the study. Your participation is voluntary. You have the right to decline, or withdraw from the study before submission of participation form. There will be neither direct benefit to participants or organization, nor any harm as a result of your participation. The data you provide will remain confidential, and will not be used except for this research purposes.

□ Agree to participate

□ Disagree

➢ **Please answer all questions:**

0- Participant's Personal Data:

0-1 Participant’s Country:

☐ United Arab Emirates ☐ Bahrain ☐ Saudi Arabia

☐ Oman ☐ Qatar ☐ Kuwait

0-2 Age:

☐ Less than 30 years ☐ 30-39 years ☐ 40-49 years

☐ 50-59 years ☐ 60+ years

0-3 Gender: ☐ Male ☐ Female

0-4 Employer’s Type: ☐ Government ☐ Non-Government

0-5 Employer’s Scope of Work: ☐ Local ☐ National

0-6 Employer’s Sector: ☐ Health ☐ Social

☐ Law enforcement / Judicial

☐ Other: ………………

Dimension 1: Trends of Violence:

1-1 How would you estimate the change in number of cases of violence against children registered with you during the pandemic?

☐ Increased, please specify percentage:

☐ Less than 10%   ☐ 10-25%   ☐ 26-50%   ☐ More than 50%

☐ Decreased, please specify percentage

☐ Less than 10%   ☐ 10-25%   ☐ 26-50%   ☐ More than 50%

☐ No change observed

1-2 Was the change:

☐ General   ☐ In a specific form(s) of violence, please specify:

☐ Political

☐ Sexual

☐Emotional

☐ Neglect

1-3 How would you estimate change in the severity of cases of violence against children recorded during the pandemic?

☐ A noticeable increase   ☐ A slight increase   ☐ Simple decrease

☐ Significant decrease   ☐ Not changed from before the pandemic

1-4 Was the change:

☐ General ☐ In a specific form(s) of violence, please specify:

☐ Political

☐ Sexual

☐Emotional

☐ Neglect

Dimension 2: Reporting and Documentation:

2-1 How did the pandemic affect your reporting mechanisms of violence against children?

☐ Positive Impact ☐ Negative Impact ☐ Both ☐ None

List the pros, if any:

1-

2-

3-

4-

List the cons, if any:

1-

2-

3-

4-

2-2 Did your reporting mechanisms of violence against children stop during the pandemic?

☐ Completely ☐ Temporary ☐ Never stopped (refer to Q2-4)

2-3 Which of your reporting mechanisms were affected by the pandemic?

No.	Affected mechanism	Totally affected	Partially affected	Reason
1		☐	☐	
2		☐	☐	
3		☐	☐	
4		☐	☐	

2-4 Have you put in place reporting mechanisms during the pandemic?

☐ No ☐ Yes, mention them: ………………………………………………………………..

2-5 How did the pandemic affect your mechanisms / records for documenting violence against children??

☐ Positive Impact ☐ Negative Impact ☐ Both ☐ None

List the pros, if any:

1-

2-

3-

4-

List the cons, if any:

1-

2-

3-

4-

2-6 Did your mechanisms / records for documenting violence against children stop during the pandemic?

☐ Completely ☐ Temporary ☐ Never stopped (refer to Q2-4)

2-7 What mechanisms / records for documenting violence against children have been affected by the pandemic?

No.	Affected mechanism	Totally affected	Partially affected	Reason
1		☐	☐	
2		☐	☐	
3		☐	☐	
4		☐	☐	

2-8 Did you create mechanisms / documentation records for violence against children during the pandemic? -

☐ No ☐ Yes, mention them: ………………………………

Dimension 3: Response and Follow-up:

3-1 How did the pandemic affect response and follow-up mechanisms for cases of violence against children?

☐ Positive Impact ☐ Negative Impact ☐ Both ☐ None

List the pros, if any:

1-

2-

3-

4-

List the cons, if any:

1-

2-

3-

4-

3-2 Did your response and follow-up mechanisms for violence against children have stopped during the pandemic?

☐ Completely ☐ Temporary ☐ Never stopped (refer to Q3-4)

3-3 What are the mechanisms of response and follow-up to your cases of violence against children that have been affected by the pandemic?

No.	Affected mechanism	Totally affected	Partially affected	Reason
1		☐	☐	
2		☐	☐	
3		☐	☐	
4		☐	☐	

3-4 Have you developed response and follow-up mechanisms during the pandemic?

☐ No ☐ Yes, mention them: ……………………………………………………………..

Dimension 4: Procedures and Legislations:

4-1 During the pandemic, were there any procedures or legislations introduced to deal with violence against children?

☐ Yes, list them:

No.	Procedures or legislations	Scope		
		Nationa<br>l	Loca<br>l	Institutiona<br>l
1		☐	☐	☐
2		☐	☐	☐
3		☐	☐	☐
4		☐	☐	☐

☐ No, no procedures or legislation has been introduced.

4-2 During the pandemic, has any procedure or legislation dealing with violence against children been withheld?

☐ Yes, list them:

No.	Procedures or legislations	Scope		
		Nationa<br>l	Loca<br>l	Institutiona<br>l
1		☐	☐	☐
2		☐	☐	☐
3		☐	☐	☐
4		☐	☐	☐

☐ No, no procedures or legislation has been withheld.

4-3 During the pandemic, was there any procedure or legislation that you believe it contributed to an increase in violence against children?

☐ Yes, list them:

No.	Procedures or legislations	Scope		
		Nationa<br>l	Loca<br>l	Institutiona<br>l
1		☐	☐	☐
2		☐	☐	☐
3		☐	☐	☐
4		☐	☐	☐

☐ No, no procedures or legislation has been introduced.

Dimension 5: Response and Follow-up:

5-1 Were there any material resources allocated to child protection in your agency affected by the pandemic?

☐ Positive Impact ☐ Negative Impact ☐ No Impact (refer to Q5-3)

5-2 What is the percentage of this change (if any):

- Increased □ less than 25% □ 25-49% □ 50-74% □ 75+%
- Decreased □ less than 25% □ 25-49% □ 50-74% □ 75+%

5-3 What are the sources of funding for your organization?

☐ Government ☐ Non-Government ☐ Both

5-4 Have the human resources working in child protection in your agency been affected by the pandemic?

☐ Positive Impact ☐ Negative Impact ☐ No Impact (refer to Q6-1)

5-5 What is the percentage of this change (if any):

- Increased □ less than 25% □ 25-49% □ 50-74% □ more than 75%
- Decreased □ less than 25% □ 25-49% □ 50-74% □ more than 75%

Dimension 6: Staff Preparation and Work Environment:

6-1 During the pandemic, did your organization convert the work environment to a virtual (remote work)?

☐ Completely ☐ Partially ☐ Never switched to remote work

6-2

**Has your organization provided child protection workers with preventive precautions to protect against Infection**?	Never	Rarely	Sometimes	Often	Always	NA
	☐	☐	☐	☐	☐	☐

6-3

**Has your organization provided child protection workers with the necessary technical equipment to work in light of the pandemic (such as Computers, communication systems, access to databases)?**	Never	Rarely	Sometimes	Often	Always	NA
	☐	☐	☐	☐	☐	☐

6-4

**Did your organization provide workers with training to deal with cases of violence against children during the pandemic?**	Never	Rarely	Sometimes	Often	Always	NA
	☐	☐	☐	☐	☐	☐

6-5

**Has your organization provided training for child protection workers to overcome the pressures of working in light of the pandemic?**	Never	Rarely	Sometimes	Often	Always	NA
	☐	☐	☐	☐	☐	☐

6-6

**Have you set up mechanisms to act in light of future pandemics / disasters?**	Never	Rarely	Sometimes	Often	Always	NA
	☐	☐	☐	☐	☐	☐

Dimension 7: Preventive Programs:

7-1 During the pandemic, did your organization present any preventive programs to the community on child protection?

☐ Yes, list them:

No.	Programs	Scope		
		National	Local	both
1		☐	☐	☐
2		☐	☐	☐
3		☐	☐	☐
4		☐	☐	☐

☐ No, no preventive programs were presented.

7-2 In your opinion, was there a national priority to protect children from violence during the pandemic?

☐ Yes ☐ No

7-3 In your opinion, were the efforts to prevent the child during the pandemic sufficient?

☐ Yes ☐ No

7-4 How many institutional partnerships can you enumerate that provided preventing violence against children during the pandemic:

☐ 0-3 ☐ 4-6 ☐ 7 or more

7-5 Has your organization contributed to these efforts to prevent violence against children during the pandemic?

☐ Yes ☐ No

7-6 Was there any political leaders committed to preventing violence against children during the pandemic?

☐ Yes ☐ No

7-7 Were communication efforts between individuals and institutions to prevent violence against children during the pandemic sufficient?

☐ Yes ☐ No

Dimension 8: Scientific Knowledge:

8-1 In your opinion, what are the main risk factors for violence against children during the pandemic?

1-
2-
3-
4-

8-2 In your opinion, what are the potential consequences of violence against children?

1-
2-
3-
4-

8-3 In your opinion, have the various efforts of *<u>international organizations</u>* contributed (such as lectures, scientific publications, websites, Social media sites) in raising knowledge of violence against children?

☐ Yes ☐ Yes, to some extent ☐ No

8-4 Did the various efforts of the *<u>local institutions</u>* (such as lectures, scientific publications, Electronic websites, social networking sites) contribute in raising knowledge of violence against children?

☐ Yes ☐ Yes, to some extent ☐ No

Dimension 9: Challenges and Recommendations:

9-1 What are the main challenges you faced in the field of child protection and prevention of violence against children during the pandemic?

1-
2-
3-
4-
5-

9-2 What are the main recommendations that you make for improving the quality of child protection services during the pandemic and preventive efforts during the pandemic?

1-
2-
3-
4-
5-

Thanks for your participation.
